# Obesity Correlates with Chronic Inflammation of the Innate Immune System in Preeclampsia and HELLP Syndrome during Pregnancy

**DOI:** 10.3390/biomedicines11102851

**Published:** 2023-10-20

**Authors:** Julia Rimboeck, Michael Gruber, Marco Weigl, Pia Huber, Dirk Lunz, Walter Petermichl

**Affiliations:** 1 Department of Anesthesiology, University Hospital of Regensburg, 93042 Regensburg, Germany; 2 University Department of Obstetrics and Gynecology at the Hospital St. Hedwig of the Order of St. John, University of Regensburg, 93049 Regensburg, Germany

**Keywords:** HELLP, preeclampsia, HDP, pregnancy, obesity, PMNs, neutrophils, ROS, NETosis

## Abstract

HELLP syndrome is characterized by hemolysis, elevated liver enzymes, and a low platelet count and poses an increased risk to the pregnant woman and the unborn child. Individual risk factors such as obesity may alter immunocompetence and influence the course of preeclampsia (PE) or HELLP syndrome. Blood samples were collected from 21 pregnant women (7 healthy, 6 with PE, and 8 with HELLP syndrome) and polymorphonuclear neutrophils (PMNs) were subsequently isolated. Production of radical oxygen species (ROS), cell movement, and NETosis were assessed by live-cell imaging. Surface protein expression and oxidative burst were analyzed by flow cytometry. PE and HELLP patients had significantly higher BMI compared to the healthy control group. Depending on the expression of CD11b, CD62L, and CD66b on PMNs, a surface protein activation sum scale (SPASS) was calculated. PMNs from patients with high SPASS values showed prolonged and more targeted migration with delayed ROS production and NETosis. Obesity is associated with a chronic inflammatory state, which in combination with immunological triggers during pregnancy could modulate PMN functions. Pregnant women with higher BMI tend to have higher SPASS values, indicating activation of the innate immune system that could co-trigger PE or HELLP syndrome.

## 1. Introduction

HELLP syndrome and preeclampsia (PE) are severe variants of hypertensive diseases of pregnancy (HDPs). HELLP syndrome has an overall incidence in all pregnancies between 0.2% and 0.8%, and PE occurs simultaneously in 70–80% of HELLP syndrome cases [1,2].

The combination of hemolysis, elevated liver enzymes, and low platelet count characterize HELLP syndrome. It can lead to life-threatening complications for both the mother and the fetus [3]. The pathogenesis of HELLP syndrome is complex and still not completely understood [4]. 

It is known that HELLP syndrome is likely caused by inadequate remodeling of spiral arteries and trophoblast invasion leading to apoptosis and necrosis, followed by release of anti-angiogenic factors and a systemic inflammatory response [5,6]. Compared to a mild systemic inflammatory response in a healthy pregnancy, HELLP syndrome is also associated with severe leukocytosis, increased levels of pro-inflammatory cytokines, and decreased levels of anti-inflammatory cytokines [7].

The innate immune system with its cells capable of phagocytosis is the first player in inflammatory responses. Polymorphonuclear neutrophil granulocytes (PMNs) represent the largest number of phagocytic cells and play an important role in defense against infections with an enormous production rate of 10^11^ cells per day [8]. In case of inflammation, PMNs release pro-inflammatory mediators, interact with and modulate other cells like lymphocytes or platelets [9,10,11,12], and cross the endothelial barrier along a chemotactic gradient toward the site of inflammation [13]. However, when misguided or overactive, PMNs can severely damage endogenous tissues [14,15,16].

Potential immunological risk factors for the development of PE or HELLP syndrome [17] and whether an inflammatory stimulus of these conditions, likely triggered by the placenta [18,19,20,21], affects the innate immune system, including PMNs, are still under investigation [22]. Therefore, the aim of this study was to characterize PMN functions in a healthy pregnancy compared with expectant mothers with PE or HELLP syndrome in terms of PMN activation—upregulation of CD11b and CD66b [23], downregulation of CD62L [24], altered chemotactic migration, and variation in the time course of reactive oxygen species (ROS) production and NETosis [25].

## 2. Materials and Methods

The Ethics committee of the University of Regensburg (Germany) approved this study (case number: 19-1270-101). 

### 2.1. Study Plan

**Figure 1:**
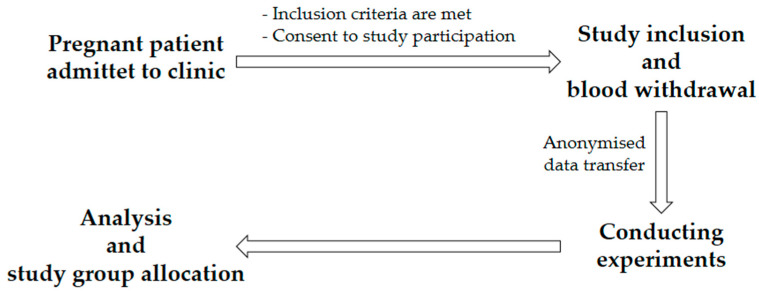
Procedure for patient inclusion in the study.

Patient recruitment took place over a period of 2 years (Figure 1). The following inclusion criteria were applied to the control group: existing pregnancy (at least 25 weeks of pregnancy), age of at least 18, voluntary written consent, and no presence of PE or HELLP syndrome. The following conditions were applied to the PE or HELLP group: existing pregnancy (at least 25 weeks of pregnancy), age of at least 18, voluntary written consent, and occurrence of PE or HELLP syndrome. The exclusion criteria for all three study groups were an age under 18, patient refusal, withdrawal of consent, known anemia, or emergency indication for caesarean section. Because this was an ex vivo study with no diagnostic implications and not an intervention study, there were no discontinuation criteria. 

**Figure 2:**
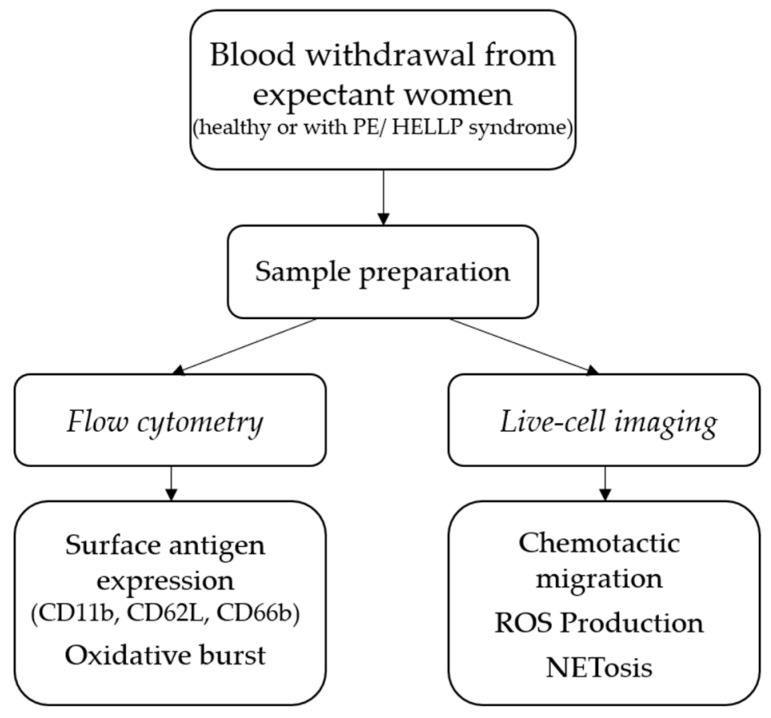
Schematic presentation of the methods used in this study.

To address the question of whether the characteristics of blood PMNs differ between healthy pregnant women and those with PE or HELLP syndrome, live-cell imaging and flow cytometric analyses were performed (Figure 2). Apart from the physician who took the blood samples, all investigators were unaware of the health status of the patients until the end of analysis.

### 2.2. Blood Withdrawal and PMN Isolation

After admission to the clinic, blood was drawn from the pregnant women for clinical purposes. Laboratory parameters (blood count, coagulation status, clinical chemistry, and partial sFlt-1/PlGF ratio) were measured according to in-house standards. Laboratory results and patient data (age, height, weight, time of pregnancy, etc.) were obtained from the medical record and anonymized before analysis. 

The experimental setup was based on previous studies of our laboratory [26,27]. Briefly, whole blood was drawn from 21 pregnant women after informed consent and anticoagulated with lithium heparin. PMNs were isolated by density gradient centrifugation (756× *g*) with Lympho and Leuko Spin Medium (pluriSelect Life Science, Leipzig, Germany) according to the manufacturer’s instructions. Cells for flow cytometric analysis were taken directly from the PMN ring layer. PMNs for microscopic analysis were previously washed in phosphate-buffered saline (PBS with Ca/Mg; Sigma-Aldrich, St. Louis, MO, USA).

### 2.3. Flow Cytometry

#### 2.3.1. Surface Antigen Expression

The expression of surface antigens was examined by immunostaining with PE-conjugated anti-CD11b (ICRF44; BioLegend, San Diego, CA, USA), FITC-conjugated anti-CD62L (DREG65; BioLegend), and APC-conjugated anti-CD66b (G10F5; BioLegend). For this purpose, 20 µL of cell concentrate was mixed with 1 mL PBS and centrifuged at 425× *g* for 3 min. The supernatant was removed and 5 μL each of CD11b, CD62L, and CD66b anti-human antibodies were added and incubated at 4 °C for 15 min. The cell suspension was washed with 2 mL PBS; after centrifugation, the supernatant was removed again, and the cells were resuspended (200 µL PBS) and analyzed. 

#### 2.3.2. Oxidative Burst

The materials and methods used to quantify ROS production have been described in detail in previous publications of our department [22,23]. Briefly, cells were pre-incubated in 500 μL PBS (without Ca/Mg; Sigma-Aldrich), 5 μL dihydrorhodamine 123 (DHR; 10 μM, Thermo Fisher Scientific, Waltham, MA, USA), and 5 μL seminaphtharhodafluor (SNARF; 10 μM, Invitrogen, Thermo Fisher Scientific). Burst was stimulated by N-formylmethionyl-leucyl-phenylalanine (fMLP; 10 μM, Sigma-Aldrich) in combination with tumor necrosis factor alpha (TNFα; 1 μg/mL, Pepro-Tech Inc., Rocky Hill, NJ, USA) or with phorbol-12-myristate-13-acetate (PMA; 10 μM, Sigma-Aldrich). Propidium iodide (PI; 1.5 µM, Thermo Fisher Scientific) was added to detect dead cells. 

#### 2.3.3. Flow Cytometric Measurement and Software Data Analysis

Flow cytometric analyses were performed using a FACSCalibur™ flow cytometer (BD Corp., Franklin Lakes, NJ, USA) and CellQuest Pro Software™ (version 5.2, BD Corp.). PMNs were identified by their typical patterns displayed in forward-scattered light and side-scattered light. Data were subsequently analyzed using FlowJo™ analysis software (version 10.0.7, BD Corp.). 

To investigate NETosis, ROS production, and migration behavior of PMNs with increased or decreased CD11b, CD62L, and CD66b surface proteins, a categorization of these molecules had to be introduced. The discriminatory values for CD patterns in flow cytometry were determined by calculating the mean fluorescence values and adding the standard deviations (SD) for CD11b and CD66b and by the mean value and subtracting SD for CD62L of the healthy control group. 

### 2.4. Live-Cell Imaging

#### 2.4.1. Experimental Setup

**Figure 3:**
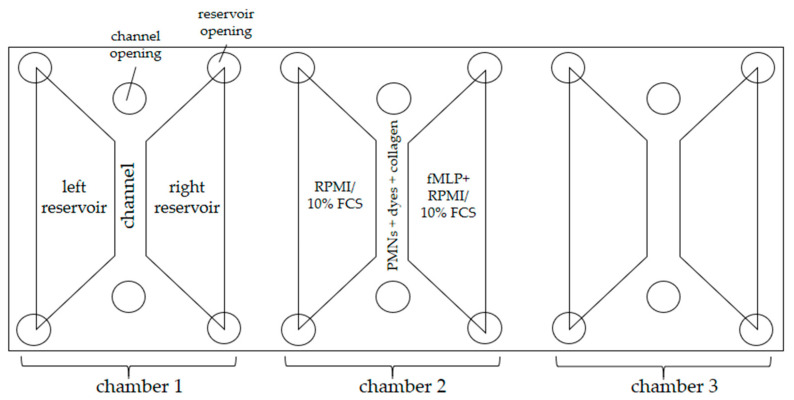
Schematic representation of the 3D-µ-Slide used; RPMI = Roswell Park Memorial Institute 1640 medium, FCS = fetal bovine serum.

To observe PMN migration, NET formation, and ROS production, 3D-µ-Slide chemotaxis chambers (Ibidi GmbH, Graefelfing, Germany) were used. One slide consists of three separate chambers, which have a channel in the middle and a reservoir to the right and left of each (Figure 3). 

The right reservoirs were filled with fMLP and RPMI 1640 (PAN-Biotech GmbH, Aidenbach, Germany)/10% FCS (fetal bovine serum; Sigma-Aldrich) and the left ones with RPMI/10% FCS without fMLP to establish a linear gradient of the chemoattractant. ROS production was visualized by oxidation of DHR 123 to fluorescent rhodamine 123. NET formation was visualized using the DNA stain 4′,6′-diamidino-2-phenylindole (DAPI; Sigma-Aldrich) and an APC-conjugated anti-human-myeloperoxidase antibody (MPO; Miltenyi Biotec, Bergisch Gladbach, Germany). The composition of the channel and reservoir fillings are listed below (Table 1).

Live-cell imaging was performed with a Leica DMi8 microscope with an automatically adjustable microscope stage and a Leica camera (DFC9000). To ensure stable test conditions (37 °C, 5% CO_2_), a stage-top incubator (Ibidi GmbH) was used. During the observation period of 6 h, fluorescence and phase contrast images were taken automatically every 30 s by the Leica Application Suite X (version 3.0.4.16529; all Leica supplies from Leica Microsystems GmbH, Wetzlar, Germany). 

#### 2.4.2. Chemotactic Migration

PMNs migrated towards an fMLP gradient from the channel in the direction of the right reservoirs. We investigated the following migratory parameters (Table 2):

To exclude artefacts, a TL ≥ 25 µm (about twice the diameter of a PMN) and simultaneously a track duration ≥ 900 s were used. The analysis of the image data was based on previous studies [27]. Briefly, a total of 720 image series per channel were analyzed using Imaris software (version 9.0.2, Bitplane, Zurich, Switzerland). To evaluate cell migration tracks, the software detected and tracked migrating cells semi-automatically for 1 h in two 30 min time periods or over an observation period of 1–4 h. The data were then exported to Excel (Microsoft Corp., Redmond, WA, USA).

#### 2.4.3. ROS Production, NET Formation, and MPO Release

During a period of 6 h, surfaces (spots [µm^2^] of ROS, DAPI, and MPO stained areas) were observed. The areas from each staining, recorded at the same time point, were summed using Excel. Detection of ROS production using DHR 123 resulted in a parabolic curve, when the sum of areas per time point was plotted against time. The time to maximum ROS production (T_max_ROS (min)) was determined using a 3° polynomial trend line of the corresponding section of the graph. The first derivative of this formula and the zero point of the latter were calculated. Time-resolved DNA or MPO release resulted in sigmoidal curves when the sum of areas per time point was plotted against time [26]. The time to half-maximum effect values (ET_50_NETs (min) and ET_50_MPO (min)) was calculated with Phoenix™ Software (Certara Inc., Princeton, NJ, USA) using E_max_ models for each staining. Extrapolated ET_50_ values far beyond the observation period (ET_50_NETs/MPO > 600 min) were excluded.

### 2.5. Statistical Evaluation

All Excel data were then transferred to SPSS^®^ Statistics (version 28.0.1.1, IBM Corp., Armonk, NY, USA), and Kolmogorov–Smirnov tests were used to confirm normal distribution for each group. When a normal distribution was present, means were compared using one-way analysis of variance. Values that are not normally distributed were compared with the Kruskal–Wallis ANOVA. In case of variance homogeneity (Levene test), post hoc analysis was performed using the Bonferroni correction. These data are presented as medians (box plots with 25–75% percentiles). *p* values less than or equal to 0.05 were considered statistically significant. Small circles in the graphics indicate statistical outliers (≥1.5 × IQR) and asterisks display extreme values (≥3 × IQR). 

## 3. Results

### 3.1. Demographical and Clinical Characteristics

Between January 2019 and February 2021, 21 pregnant women (7 healthy, 6 with PE, and 8 with HELLP syndrome) were enrolled in this study (Table 3). At the time of blood collection, the women were on average 33 weeks ± 4 days pregnant. The mean age of the women was 33 ± 4 years with a range of 26 to 41 years. There were no differences in age, height, or duration of pregnancy between the study groups. Pregnant women with HELLP syndrome or PE had significantly higher body weight and body mass index (BMI) compared with healthy pregnant women. HELLP patients had no differences in weight or BMI compared with those with PE. 

In our study, all women with PE or HELLP syndrome had a preterm birth before the 37th week of pregnancy (14/14; 100%). No preterm birth was recorded in the group of healthy pregnant women (0/7; 0%; *p* < 0.001). Two patients from the HELLP group required admission to the intensive care unit. PE patients in our study had a significantly higher need for antihypertensive medication compared to healthy pregnant women (100% vs. 0%; *p* < 0.001). Patients with HELLP syndrome also needed significantly more antihypertensive medication than healthy pregnant women (75% vs. 0%; *p* < 0.001). All pregnant women with hypertension were treated with alpha–methyldopa, an alpha-2-adreno-receptor agonist. Two expectant women from the HELLP group and two from the PE group were additionally treated with Nifedipine, a calcium-receptor antagonist. Glucocorticoids were given to 88% (7/8) of the pregnant women with HELLP syndrome. No healthy pregnant women and no PE patients were treated with glucocorticoids (*p* < 0.001). Patients in the healthy group (no PE, no HELLP syndrome) received low-molecular-weight heparin due to cervical insufficiency. No patient with PE but three women with HELLP syndrome received heparin. 

### 3.2. Platelets, Leukocytes, CRP, GPT, GOT, and Creatinine

The mean values of standard clinical chemistry parameters showed significant differences between the healthy expectant women and those with HELLP syndrome (Table 4). Only when observing the leukocyte counts did values from PE patients differ from the two other groups.

### 3.3. sFlt-1/PlGF Ratio

In this study, HELLP patients did not have a significantly increased anti-angiogenic soluble fragment of the VEGF receptor (sFlt-1) compared to pregnant women with PE (*p* = 0.086). Moreover, PE patients had no difference in placenta growth factor values (PlGF) compared to pregnant women with HELLP syndrome (*p* = 0.290) (Table 5). 

Based on standard values from the literature, the sFlt-1/PlGF ratio was considered normal when the cutoff of 38 was not exceeded [28,29,30]. All patients in the PE and in the HELLP group had a pathological sFlt-1/PlGF ratio. The sFlt-1/PlGF ratios of pregnant women with PE were not different from pregnant women with HELLP (*p* = 0.424) (Table 5). Only one pregnant woman from the healthy group was analyzed for the sFlt-1/PlGF ratio. This woman had no pathological increased sFlt-1/PlGF ratio (value < 10).

### 3.4. Expression of Surface Antigens CD11b, CD62L, CD66b, and SPASS Index

PMNs from patients with HELLP syndrome showed increased expression of CD11b and CD66b and a decreased expression of CD62L compared to the group of healthy pregnant women. PMNs from PE patients showed higher CD66b expression and significantly lower CD62L expression (Table 6).

Linear regression analyses of BMI and the expression of CD11b showed no correlation (R² = 0.018), the expression of CD62L showed an inverse correlation (R² = 0.483), and the expression of CD66b showed a weak positive correlation (R² = 0.110).

#### Introduction of Surface Protein Activation Sum Scale (SPASS) 

A new classification system for antigen expression of PMNs was introduced. For this purpose, the following discrimination levels of fluorescent intensities (afu) were used: CD11b^high^ > 512; CD62L^low^ < 106; and CD66b^high^ > 223. 

Using the classification CD11b^high^ = 1 and CD11b^low^ = 0; CD62L^high^ = 0 and CD62L^low^ = 1; as well as CD66b^high^ = 1 and CD66b^low^ = 0, a surface protein activation sum scale (SPASS) of PMNs could be calculated. The values ranged from 0 (all surface proteins at a non-activated level) to 3 (all surface protein expressions activated) and could be assigned to the health status of the expectant women (Table 7).

**Figure 4:**
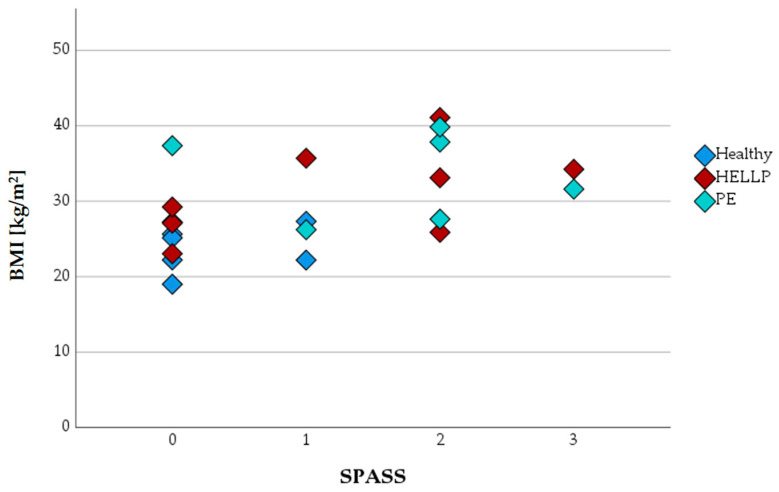
SPASS values of PMNs in healthy pregnant women and in PE or HELLP syndrome depending on the patients’ BMI.

When SPASS values were plotted against the BMI, healthy expectant women were found only in the SPASS groups 0 or 1 (Figure 4). 

### 3.5. Oxidative Burst

Mean values of median ROS intensities in the healthy/PE/HELLP study groups were compared after receptor-mediated activation (with fMLP + TNFα) or direct protein kinase C activation (with PMA) (Table 8). 

Overall, activation with PMA resulted in higher fluorescence intensities, whereas receptor activation resulted in lower activation. There was no statistically significant difference in the mean values of median ROS intensity after stimulation with fMLP + TNFα with respect to the health status (F (2,16) = 0.819, *p* = 0.459, η^2^ = 0.093) or the SPASS values (F (3,15) = 0.780, *p* = 0.523, η^2^ = 0.135). Moreover, no significant differences in ROS intensities were detected after stimulation with PMA in relation to different health status (F (2,17)= 0.104, *p* = 0.902, η^2^ = 0.012) or the SPASS values (F (3,16) = 0.635, *p* = 0.603, η^2^ = 0.106). 

Independently of the health status or the SPASS values, the portion of dead PMNs was at a level of 1.12 ± 0.86%. 

### 3.6. Chemotactic Migration

#### 3.6.1. Progression of TL as a Function of Health Status or SPASS Values

**Figure 5:**
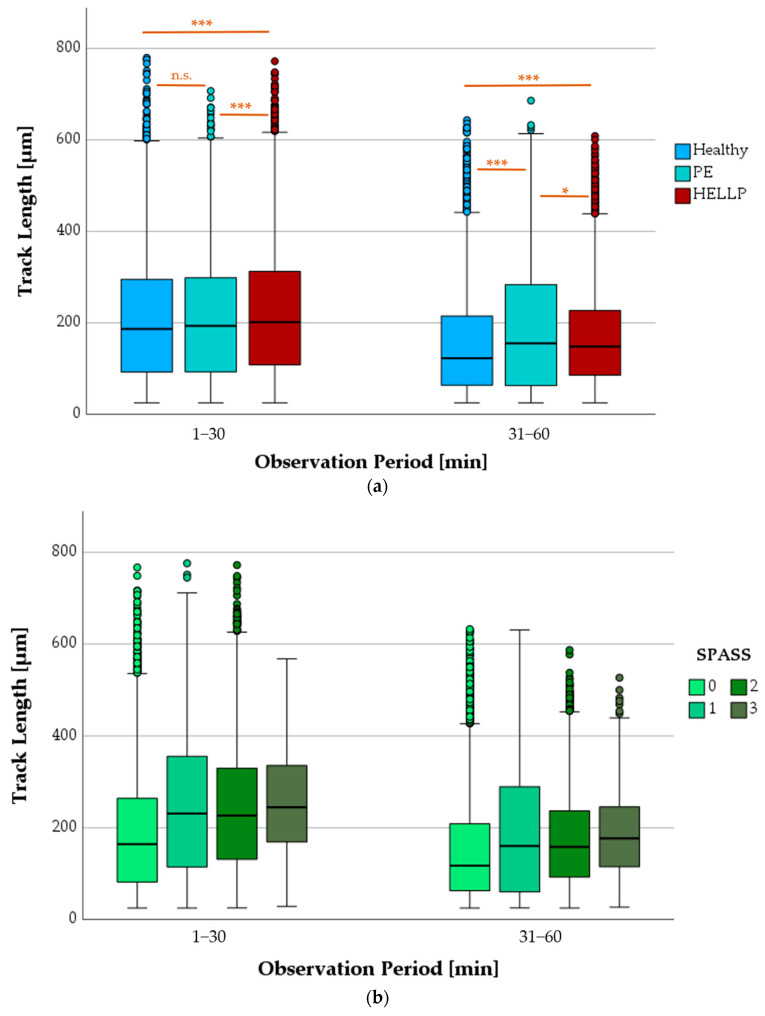
Comparison of PMN TL with respect to the observation period and the health status (**a**) or the SPASS values (**b**); n.s. means not significant, * *p* < 0.05, *** *p* < 0.001; small circles in graphics mark statistical outliers (≥1.5 × IQR).

Observation of PMN tracks according to health status showed between 983 and 9035 tracks per box and a median length of 195 µm during the first 30 min and 140 µm between 31 and 60 min after starting the microscope. During both observation periods, a significant difference in TL was always observed between the HELLP and the healthy groups (*p* < 0.001). The TL of the PE patients and the healthy group differed significantly only in the observation period of 31–60 min (*p* < 0.001) (Figure 5a). 

Depending on the increase in the SPASS value, a more pronounced boost in migration, represented by the TL, could be observed (Figure 5b). During both time periods, the TL of PMNs with SPASS = 0 were significantly different from all others (SPASS values 1 to 3) (*p* < 0.001). At SPASS = 1, the TL were comparable to those of the SPASS 2 group—no significant differences were seen. The TL values of the SPASS 3 group were significantly higher than those of all other groups in both observation periods (*p* < 0.001).

#### 3.6.2. Progression of Track Straightness as a Function of SPASS Values

**Figure 6:**
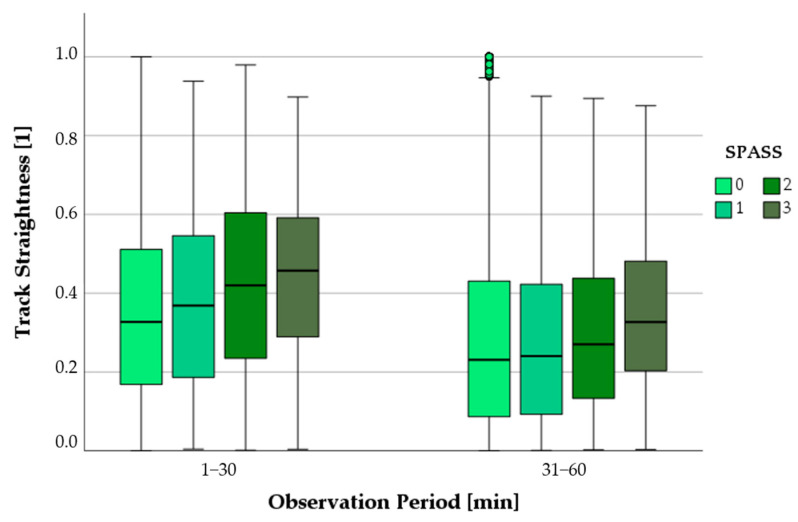
Comparison of track straightness of PMNs depending on the SPASS index over the course of the first hour; small circles in graphics mark statistical outliers (≥1.5 × IQR).

As the SPASS values increased, the straightness of the tracks also increased (Figure 6). In the first half hour, the track straightness of PMNs was significantly lower at SPASS values of 0 compared to all other SPASS values (1–3) (*p* < 0.001). In the next observation period (31–60 min), the straightness values of the SPASS group 0 were not significantly different from the values at SPASS 1, but they were significantly lower than those at SPASS values of 2 or 3 (*p* < 0.001). 

#### 3.6.3. Progression of TL as a Function of Antigen Expression State

**Figure 7:**
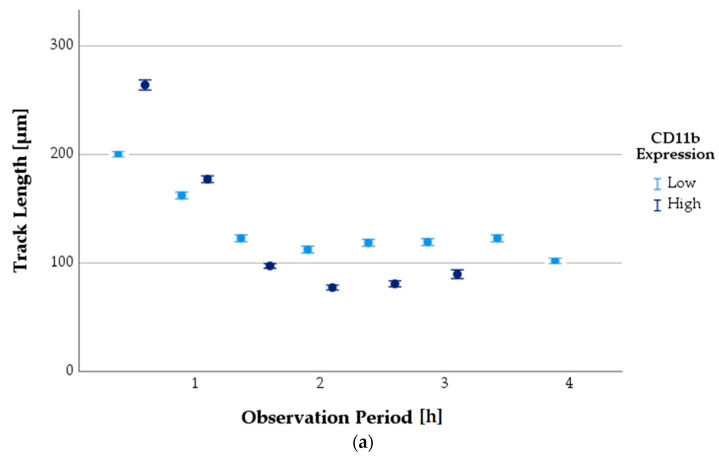

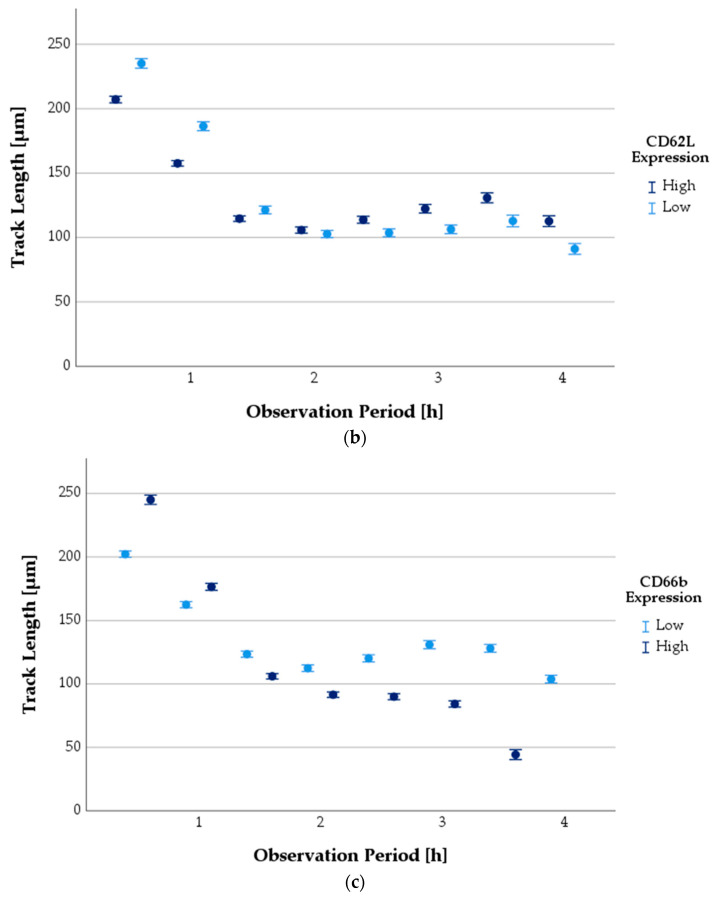
Comparison of mean PMN TL over the observation period depending on surface antigen expression status (error bars: 95% CI). TL depending on expression of CD11b (**a**), CD62L (**b**), and CD66b (**c**).

The TL of PMNs could also be compared over the observation period as a function of the surface antigen expression state (Figure 7). When PMNs were in an activated state, the surface antigens CD11b and CD66b were more highly expressed, whereas CD62L was less expressed. 

Activated PMNs had a longer TL at baseline than non-activated ones. After an observation period of approximately 1.5 to 2 h, a reversal was observed: non-activated PMNs showed a longer TL from then until the end of the observation. 

### 3.7. ROS Production

#### 3.7.1. The Progress of the Average ROS Intensity along the Observation Period

**Figure 8:**
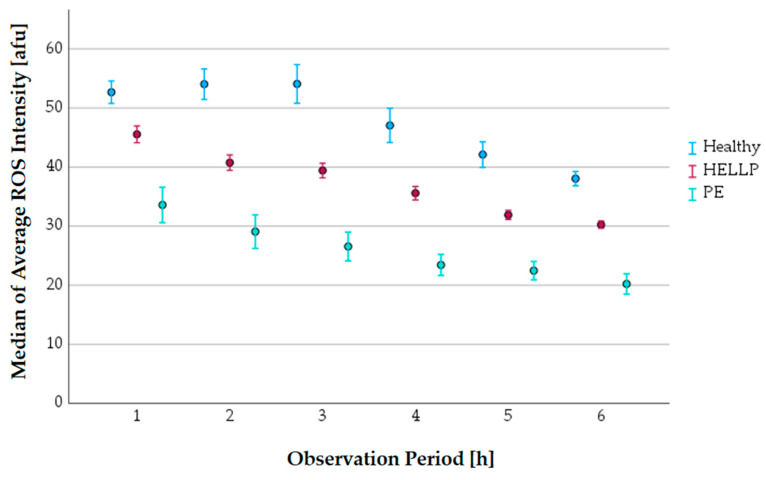
The progress of the average ROS intensity along the observation period in relation to the health status (error bars: 95% CI).

PMNs can produce ROS. The median of the average ROS intensity was analyzed in 1 h observation periods in relation to the health status of the pregnant women (Figure 8). 

The average ROS intensity was highest in the healthy control group and lowest in patients with PE. The values of the HELLP patients were in the middle range (Table 9). 

#### 3.7.2. T_max_ROS Depending on Health Status or SPASS Value

The number of PMNs carrying rhodamine 123 (an oxidation product of DHR) above the threshold was lower at the beginning of microscopic observation, increased with time, and then decreased. The time point of the greatest number of ROS-positive PMNs was defined as T_max_ROS (min). 

**Figure 9:**
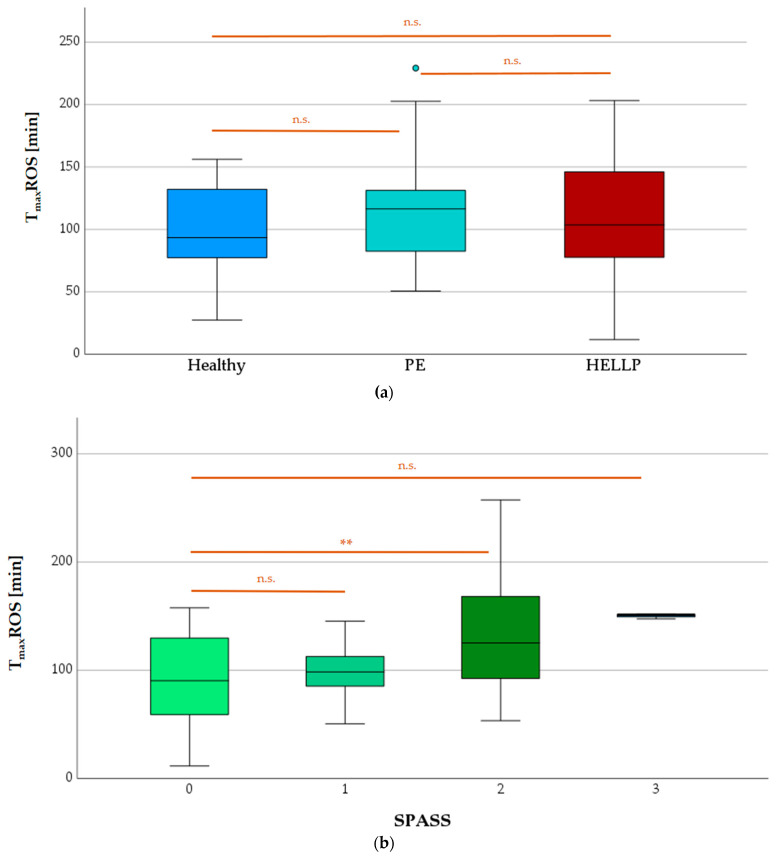
T_max_ROS depending on the health status of the pregnant women (**a**) or the SPASS values (**b**); n.s. means not significant, ** *p* < 0.01; small circles in graphics mark statistical outliers (≥1.5 × IQR).

No statistically significant differences were detected when comparing T_max_ROS means from expectant women in the three health categories (Figure 9a). With respect to the SPASS factor (Figure 9b), significant differences could be seen only between the T_max_ROS values of the women without surface protein activation (SPASS = 0) and SPASS = 2 (*p* = 0.003). 

### 3.8. NETs and MPO Release

#### 3.8.1. ET_50_NETosis in Terms of Health Status and SPASS Value

**Figure 10:**
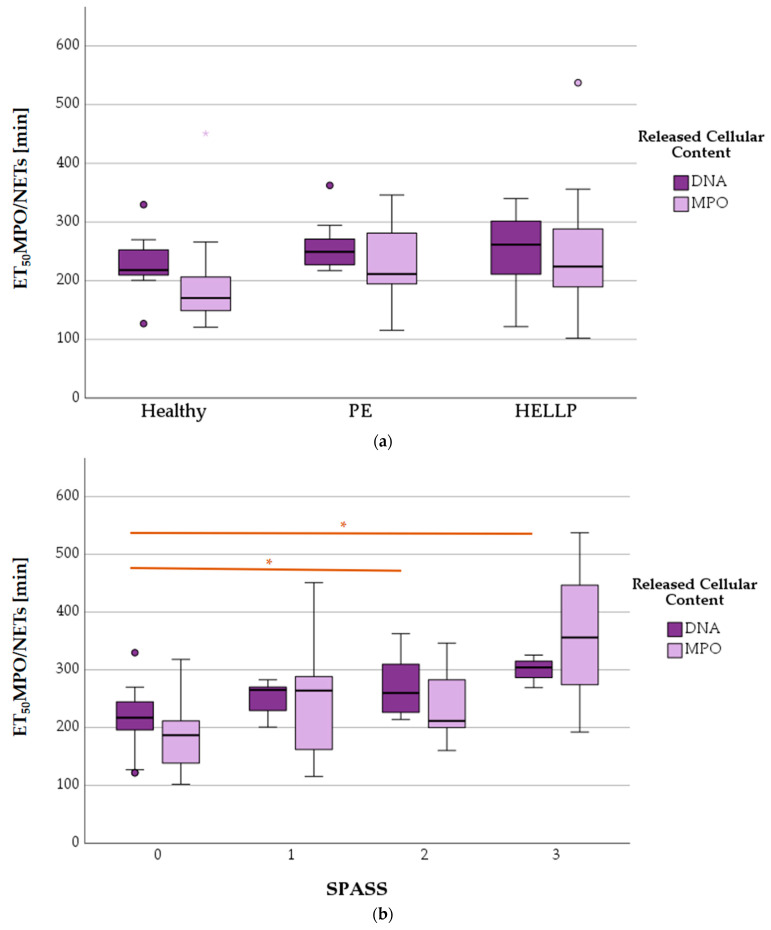
ET_50_NETosis (ET_50_MPO and ET_50_NETs) versus health status (**a**) and SPASS factor (**b**). Significant differences within the groups are marked as * (*p* < 0.05), small circles in graphics mark statistical outliers (≥1.5 × IQR).

Two cellular contents were observed during NETosis (Figure 10). The time-resolved release of MPO showed no differences depending on the health status (*p* = 0.117), but it did show differences depending on the SPASS factor (*p* = 0.032). ET_50_NETs as the calculated parameter for the DNA release also did not change significantly in the health status comparison (*p* = 0.162), but it did change significantly in the SPASS comparison (*p* = 0.003).

#### 3.8.2. Mean ET_50_NETosis Depending on PMNs’ CD11b Surface Expression

**Figure 11:**
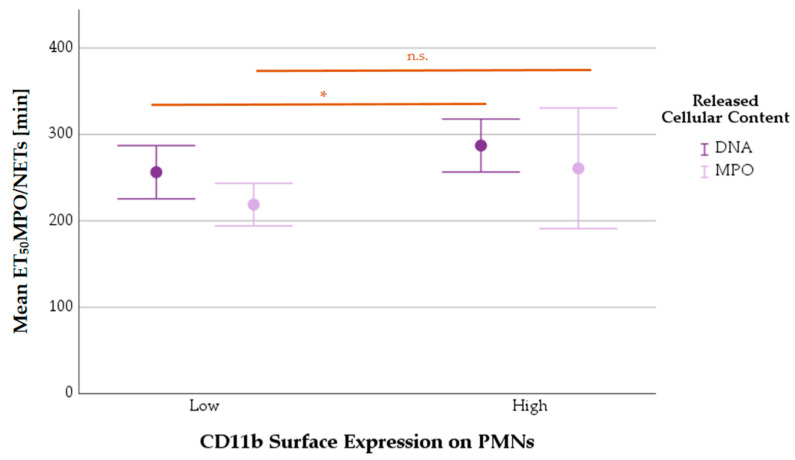
Mean ET_50_NETosis values of PMNs with low or high expressions of CD11b (error bars: 95% CI). Differences between groups are highlighted by n.s. (= statistically not significant) or * (*p* < 0.05).

The mean ET_50_NETs value was statistically significantly higher in activated PMNs (MV CD11b^high^: 287.1 ± 45.6) than in non-activated (MV CD11b^low^: 256.3 ± 103.6) ones (*p* = 0.045). The mean ET_50_ value for MPO release was not different between the two groups (MV CD11b^low^: 218.8 ± 77.1, MV CD11b^high^: 260.8 ± 109.9; *p* = 0.232) (Figure 11). 

## 4. Discussion

It is well known that the immune response changes during pregnancy [14,15]. In the last four decades, there has been a paradigm shift from immuno-suppressed to immuno-modulated pregnancies [14,16]. One way to establish immunomodulation/tolerance during pregnancy is a shift in lymphocytic defense [17]. However, not only immune tolerance but also non-infectious inflammation is necessary for a successful pregnancy. Previous studies have shown increased accumulation of phagocytic cells of innate defense (macrophages) in the placenta. These macrophages actively organize the process of tissue remodeling by contributing to blood vessel formation and cell proliferation [18]. This immune modulation, which is necessary for the well-being of the fetus, can occasionally be harmful to the mother especially in case of pregnancy-related conditions such as PE or HELLP syndrome [14]. 

While macrophages play a major role in physiological inflammatory pregnancy reactions, PMNs are the major cell types in a pathological inflammatory response, regardless of whether it is infectious or sterile [19]. PMNs show a typical pattern of their surface proteins in an inflammatory response. In sterile but also in infectious inflammation, PMNs show increased expression of CD11b [31,32,33,34], a surface protein of the integrin family, and CD66b, a surface protein of the carcinoembryonic antigen family, and a decrease in CD62L [35], a surface protein of the selectin family [20,21]. 

Flow cytometric analysis on those surface proteins (CD11b, CD62L, and CD66b) allowed us to classify PMNs into a new surface protein activation sum scale (SPASS). In this score, healthy expecting women achieved a maximum score of 1. PE or HELLP patients did not necessarily show a higher score, but they may achieve a maximum score of 3. 

In our study, we also demonstrated an increase in CD11b in HELLP patients only as well as an increased CD66b expression on PMNs in PE and HELLP patients. These results are comparable to a study by Crocker et al. in which no increased CD11b activity was detected in PE and healthy pregnant women [24]. The PMN surface protein CD62L showed a decreased expression in PE and HELLP patients. A constellation of surface molecules, CD66b^high^ and CD62L^low^ on PMNs, are evaluated in the literature as inflammatory-activated PMNs [22,23]. In our study, the decrease in CD62L on PMNs was weight-correlated. However, for the reduced expression of CD62L in patients with PE or HELLP syndrome, as found in another study of our laboratory (data not published yet), it is not the increased BMI of the patients that is the only cause but the pathological conditions themselves (PE and HELLP) may induce a reduction in the expression of CD62L in those patients with increased BMI. The proof for the correlation between the severity of PE/HELLP with BMI has to be the subject of further investigations. 

A decrease in CD62L and an increase in CD66b expression on PMNs are associated with chronic inflammation in obesity [25,26]. The decrease in CD62L and the increase in CD66b on PMNs in HELLP and PE may be partly explained by obesity-related chronic inflammation. An increase in CD11b is not known in obesity [25]. 

Chronic inflammation is partly responsible for the change in CD expression. Obesity is known to induce silent chronic inflammation; and therefore, obesity is an independent risk factor for HELLP syndrome [26]. A decreased expression of CD62L may indicate chronic inflammation in obesity during pregnancy; if a coincidence of an elevated CD11b on PMNs is also detectable, this may indicate women at risk of HELLP. 

In contrast to PE, HELLP syndrome shows increased FAS ligand activity [4]. Increased expression of the FAS ligand leads to an increased level of programmed cell death and therefore requires increased phagocytosis activity. The reduced expression of CD62L on PMNs is also indicative of increased apoptosis. A study by Matusab et al. demonstrated that a decrease in CD62L indicates aging of circulating PMNs with an increased rate of apoptosis [27]. 

CD11b promotes the activation of phagocytotic cells such as macrophages and PMNs [36]. The increased CD11b expression of PMNs in our study may indicate an adaptive reaction to an increased demand for phagocytosis. Moreover, CD11b inhibits immunosuppression, modulates neovascularization, and promotes anti-tumor immune reaction [36]. However, upregulation of CD11b is not without problems. Diabetic patients with an increased CD11b combined with a decreased CD62L often suffer from a microangiopathic disease [37]. Because microangiopathy also occurs in HELLP syndrome, the alteration of CD surface proteins of PMNs triggered by cellular damage (endothelium etc.) could be a step of pathogenesis in HELLP syndrome. There is evidence that decreased L-selectin expression leads to increased adhesion and activation of leukocytes to the damaged endothelium [38]. An increased CD11b expression is also associated with an increased adhesion effect of PMNs to fibrinogen and platelets [39,40]. 

The binding of PMNs and platelets (platelet–leukocyte complexes) leads to a non-proteolytic contact activation of complement factor 3 (C3). Activation of C3 is responsible for thrombosis with a loss of platelets and tissue damage (e.g., liver cell death with an increase in GOT, GPT) [31]. However, not only do the PMNs affect platelets but platelets also affect PMNs. Platelets inhibit the expression of CD11b and CD66b, stimulate phagocytosis, and reduce the release of elastase, so platelets exert an anti-inflammatory and tissue-protective effect on PMNs [31]. A decrease in platelet number, as found in HELLP syndrome, has an autocatalytic effect on the tissue-damaging effect of PMNs. Whether platelets bound in platelet–PMN complexes still exert their protective effect has not been adequately investigated. 

Using SPASS, we found a correlation to other PMN parameters such as migration (TL and TS), ROS production (T_max_ROS), and speed of NETosis (ET_50_MPO and ET_50_NETs). The higher the SPASS value, the higher the TL and the ability of PMNs to follow a chemotactic gradient (track straightness). 

The longer TL at the beginning of the observation period with higher SPASS values and the concomitant higher TS values suggest that more active PMNs initially have more effective migration than non-active ones, which may also allow them to arrive at the target site faster and initiate the immune response earlier. 

Activated PMNs (CD11b^high^/CD62L^low^/CD66b^high^) initially showed a longer TL than non-activated ones, but this reversed after an observation time of a maximum of 2 h. From then on, non-activated cells showed a longer migration length. NETosis of PMNs was significantly delayed at SPASS values greater than 2 and with high CD11b expression. 

Cells prone to concerted action travel longer distances [37] and infiltrate maternal systemic vasculature [38] until they must produce ROS and die at a later time point in NETosis than PMNs from healthy expectant women [39]. Nevertheless, the increased incidence of PMN cell death is likely because matrix metalloprotease 1 (MMP1), MPO, neutrophil elastase, and free DNA have been quantified at higher levels in PE patients than in controls [21,40].

Limitations: The patients with HELLP syndrome had significantly increased leukocyte counts in the peripheral blood compared to PE patients. However, seven out of eight HELLP patients received dexamethasone, which could be the cause of the increase in peripheral leukocyte count. 

Differential blood counts were not routinely performed, which is why no statement can be made about the proportion of granulocytes to total leukocytes. 

The partly different results, e.g., SPASS values of 0 or 3 within the HELLP group, could be due to strong interindividual differences in the PMN functionality of the patients. However, as the analyses were based on only one blood sample per patient, this cannot be verified in this study, as no follow-up analyses were carried out with blood samples taken at later time points.

In the entire study population, only 2 of the 21 patients (1 each with PE and HELLP syndrome) had a SPASS score of 3. 

## 5. Conclusions

During pregnancy, the immune response changes. HELLP syndrome leads to an alteration in the expression of CD markers on PMNs, similar to an inflammatory response. The upregulation of CD11b to bind to platelet factor 4 leads to the formation of platelet–PMN complexes. These aggregates lead to an increase in thrombotic microangiopathies with further tissue damage and thrombocytopenia. A mutual influence of platelets and leukocytes seems to play a crucial role in HELLP syndrome. Obesity probably leads to an amplification of the damaging effects by a chronic inflammatory response mediated by CD66b. CD11b expression is not significantly increased in PE and is independent of BMI in HELLP patients. ROS intensity is reduced in both PE and HELLP patients. 

## Figures and Tables

**Table 1:** Composition of the channel and its reservoir fillings of the 3D-µ-Slides used.

Left Reservoir (65 µL)	Channel (6 µL)	Right Reservoir (65 µL)
RPMI/10% FCS	PMNs in medium (15.7%) DAPI [0.5 µg/mL] DHR 123 [1 µM]	fMLP [10 nM] RPMI/10% FCS
	Anti-MPO antibody [0.5 µg/mL]	
	Collagen Type 1/1.67% FCS [1.5 mg/mL]	

**Table 2:** Analyzed migratory parameters, defined according Doblinger et al. [26].

Parameter (Abbreviation)	Unit	Description
Track Length (TL)	[µm]	Total length of the migration route of each individual tracked cell
Track Straightness (TS)	[1]	Fraction of Euclidean track length and total track length showing the cell’s tendency to migrate directly; higher factors refer to straighter lines

**Table 3:** Demographical characteristics: age, time of pregnancy, height, weight, and BMI of healthy pregnant women and women with PE and HELLP. All data are shown as medians (25% percentile; 75% percentile); *p* ≤ 0.05 = statistically significant; n.s. = not significant.

	Healthy	PE	*p*-Value
Age (years)	33 (28;34)	32 (30;32)	n.s.
Time of pregnancy (weeks)	33 (31;38)	35 (29;36)	n.s.
Body height (cm)	167 (158;171)	163 (161;170)	n.s.
Body weight (kg)	65 (59;71)	84 (73;107)	0.021
BMI (kg/m^2^)	22 (22;27)	32 (27;39)	0.016
	**Healthy**	**HELLP**	***p*-Value**
Age (years)	33 (28;34)	31 (29;38)	n.s.
Time of pregnancy (weeks)	33 (31;38)	33 (28;36)	n.s.
Body height (cm)	167 (158;171)	166 (163;169)	n.s.
Body weight (kg)	65 (59;71)	85 (72;100)	0.028
BMI (kg/m^2^)	22 (22;27)	31 (26;35)	0.019

**Table 4:** Comparison of clinical chemistry parameters (mean values ± SD).

	Healthy	PE	HELLP
Platelets (10^3^/µL)	232 ± 49 *p* < 0.001 vs. HELLP	182 ± 32 *p* < 0.001 vs. HELLP	80 ± 28
Leukocytes (10^3^/µL)	10.8 ± 1.9 *p* = 0.045 vs. PE	8.8 ± 1.1 *p* = 0.010 vs. PE	13.6 ± 5.5
CRP (mg/L)	4.2 ± 4.5 *p* = 0.035 vs. HELLP	3.4 ± 4.5 *p* = 0.031 vs. HELLP	32.7 ± 21.7
GPT (U/L)	16.7 ± 9.9 *p* = 0.003 vs. HELLP	19.8 ± 6.4 *p* = 0.005 vs. HELLP	191.3 ± 133
GOT (U/L)	24.3 ± 8.6 *p* < 0.001 vs. HELLP	28.7 ± 6.6 *p* = 0.001 vs. HELLP	232 ± 229
Creatinine (mg/dL)	0.70 ± 0.08 *p* = 0.009 vs. HELLP	0.70 ± 0.08 *p* = 0.012 vs. HELLP	0.95 ± 0.16

**Table 5:** Values (mean ± SD) of sFlt-1, PlGF, and the sFlt-1/PlGF ratio in patients with PE or HELLP.

	PE	HELLP
sFlt-1 [ng/mL]	12.2 ± 4.5	17.3 ± 5.4
PlGF [pg/mL]	40 ± 4	68 ± 62
sFlt-1/PlGF ratio	308 ± 98	442 ± 385

**Table 6:** Upregulation of CD11b and CD66b and downregulation of CD62L on PMNs of healthy expectant women compared to patients with PE or HELLP (mean values ± SD (afu)); *p* values comparing PE/HELLP values with healthy control group.

	Healthy	PE	HELLP
CD11b	372 ± 140	398 ± 174 *p* = 1.000	586 ± 358 *p* = 0.217
CD62L	137 ± 31.5	94.9 ± 10.6 *p* = 0.024	99.0 ± 28.8 *p* = 0.116
CD66b	176 ± 47.5	220 ± 45.4 *p* = 0.472	253 ± 84.1 *p* = 0.174

**Table 7:** SPASS values of PMN surface proteins in healthy pregnancies and in PE or HELLP syndrome.

SPASS	Healthy (*n*)	PE (*n*)	HELLP (*n*)	Total (*n*)
0	5	1	3	9
1	2	1	1	4
2	0	3	3	6
3	0	1	1	2

**Table 8:** The mean of the median ROS intensities (afu) with respect to the health status or the SPASS values.

	Healthy	PE	HELLP	
*fMLP + TNFα*	23.4 ± 20.2	25.8 ± 18.6	34.7 ± 4.9	
*PMA*	569 ± 182	553 ± 152	592 ± 129	
**SPASS**	**0**	**1**	**2**	**3**
*fMLP + TNFα*	30.1 ± 15.5	26.2 ± 22.2	20.2 ± 15.2	40.0 ± 1.4
*PMA*	620 ± 164	492 ± 93.2	570 ± 171	555 ± 123

**Table 9:** Medians of the average ROS intensity with respect to the health status.

Time [h]	Healthy	PE	HELLP
1	52.7	33.6 ***	45.5 ***
2	54.0	29.0 ***	40.7 ***
3	54.1	26.6 ***	39.4 ***
4	47.1	23.4 ***	35.6 ***
5	42.1	22.5 ***	31.9 ***
6	38.0	20.2 ***	30.3 ***

*** *p* < 0.001 comparing healthy vs. PE/HELLP.

## Data Availability

The data presented in this work are available on request from the corresponding authors.
